# Supplementary material for: Denial of legal abortion in Nepal
Source: PLoS One. 2023 Mar 21;18(3):e0282886. doi: 10.1371/journal.pone.0282886 (PMC10030013; doi:10.1371/journal.pone.0282886)
Supplement: Attachment — Submitted filename: Response to Reviwer.docx [file pone.0282886.s001.docx]

27 July 2022

Dr. Alok Atreya
Academic Editor
PLOS ONE

**Re: Revised manuscript titled ‘Denial of legal abortion in Nepal (PONE-D-22-07816)**

Dear Dr. Atreya,

Thank you very much for your very useful comments and suggestions on our manuscript. As suggested, we have revised the cover letter, checked the reference carefully including style and self-citations, and revised the manuscript accordingly. We hope that you will be happy with our revision and will proceed for the needful action.

The point-by-point response to your comments and suggestions as follows:

**Point-by-point response to comments/suggestions**

1. Please ensure that the cover letter is intended for the submission of the manuscript to Plos One.

Response: The cover letter has been revised.

2. Please check reference 13 and 14 for their completeness. I recommend the authors cited Muluki Criminal (Code) Act 2074 (2017), Paragraph 13, Article 189 for the legislature relating to abortion in Nepal. <https://www.ilo.org/dyn/natlex/docs/ELECTRONIC/106060/129899/F1095481449/NPL106060%20Npl.pdf>

Response: We have cited the most recent and relevant for this: ‘Safe Motherhood and Reproductive Health Rights Act, 2075 (2018), Chapter 4, Article 15. <https://www.lawcommission.gov.np/en/wp-content/uploads/2019/07/The-Right-to-Safe-Motherhood-and-Reproductive-Health-Act-2075-2018.pdf>

We have updated our references accordingly.

3. Regarding facilities and centers providing abortion care the authors have retrieved the information from the articles cited as references 15, 16 and 17, the latest being 2019. I recommend the authors updated the manuscript with latest current figures from the primary source (Ministry of Health and Population) rather than the cross-reference. <https://www.mohp.gov.np/eng/program/reproductive-maternal-health/nsas>

Response: Thank you – however, the suggested government citation (MOHP website) has not been updated regularly- the latest information available from the suggested site is for between 2014/15 (FY71/72). Therefore, we have used another MOHP reference to update these numbers. After 2019/2020 the government has not updated these figures in their annual report or any other publications after 2019/2020. For this, we cited the following reference

Ministry of Health and Population, Annual Report Department of Health Services 2076/77 (2019/2020). <https://dohs.gov.np/wp-content/uploads/2021/07/DoHS-Annual-Report-FY-2076-77-for-website.pdf>

4. Please ensure uniformity in the reference style.

Response: We have checked the uniformity in the reference style carefully and revised it.

5. The reference section shows too much of self-citation. Please ensure only the required and relevant articles are cited in the context to the present study. 

Responses: These are carefully checked and avoided as much as possible. We deleted 4 references including two self-citations. Moreover, we have references only that are needed and we seem self-referential because very few other research groups are studying abortion denial in Nepal and elsewhere.

Journal Requirements:

When submitting your revision, we need you to address these additional requirements.

1. Please ensure that your manuscript meets PLOS ONE's style requirements, including those for file naming. The PLOS ONE style templates can be found at 
<https://journals.plos.org/plosone/s/file?id=wjVg/PLOSOne_formatting_sample_main_body.pdf> and 
<https://journals.plos.org/plosone/s/file?id=ba62/PLOSOne_formatting_sample_title_authors_affiliations.pdf>

Response. PLoS ONE’s style has been checked while formatting our manuscript.

2. You indicated that you had ethical approval for your study. In your Methods section, please ensure you have also stated whether you obtained consent from parents or guardians of the minors included in the study or whether the research ethics committee or IRB specifically waived the need for their consent.

Response: We have added the following sentence in the method section:

“In the case of minor under 18 years of age, consent from one biological parent (either mother or father) and assent from the girl was obtained”.

3. Thank you for stating the following financial disclosure:

This study was supported by the National Institute of Health (Grant number: A133916), and the Packard Foundation (Grant Number 132968) to Diana Greene Foster, at the University of California, San Francisco. 
 Please state what role the funders took in the study.  If the funders had no role, please state: "The funders had no role in study design, data collection and analysis, decision to publish, or preparation of the manuscript." 
If this statement is not correct you must amend it as needed. 
Please include this amended Role of Funder statement in your cover letter; we will change the online submission form on your behalf.

Response: We have revised the financial disclosure as “The funders had no role in study design, data collection and analysis, decision to publish, or preparation of the manuscript”. This has also mentioned in the cover letter.

4. Thank you for stating the following in your Competing Interests section:

No authors report competing interests.

Please complete your Competing Interests on the online submission form to state any Competing Interests. If you have no competing interests, please state "The authors have declared that no competing interests exist.", as detailed online in our guide for authors at <http://journals.plos.org/plosone/s/submit-now> 
 This information should be included in your cover letter; we will change the online submission form on your behalf.

Response: This section has been updated as suggested. We have also mentioned this our cover letter.

5. In your Data Availability statement, you have not specified where the minimal data set underlying the results described in your manuscript can be found. PLOS defines a study's minimal data set as the underlying data used to reach the conclusions drawn in the manuscript and any additional data required to replicate the reported study findings in their entirety. All PLOS journals require that the minimal data set be made fully available. For more information about our data policy, please see <http://journals.plos.org/plosone/s/data-availability>.

Upon re-submitting your revised manuscript, please upload your study’s minimal underlying data set as either Supporting Information files or to a stable, public repository and include the relevant URLs, DOIs, or accession numbers within your revised cover letter. For a list of acceptable repositories, please see <http://journals.plos.org/plosone/s/data-availability#loc-recommended-repositories>. Any potentially identifying patient information must be fully anonymized.

Important: If there are ethical or legal restrictions to sharing your data publicly, please explain these restrictions in detail. Please see our guidelines for more information on what we consider unacceptable restrictions to publicly sharing data: <http://journals.plos.org/plosone/s/data-availability#loc-unacceptable-data-access-restrictions>. Note that it is not acceptable for the authors to be the sole named individuals responsible for ensuring data access.

We will update your Data Availability statement to reflect the information you provide in your cover letter.

Response: Data used in the preparation of this manuscript is uploaded at

<https://dataverse.harvard.edu/dataset.xhtml?persistentId=doi:10.7910/DVN/HMOWCA> and will be made publically available after the publication of this paper.
