# Supplementary material for: Denial of legal abortion in Nepal
Source: PLoS One. 2023 Mar 21;18(3):e0282886. doi: 10.1371/journal.pone.0282886 (PMC10030013; doi:10.1371/journal.pone.0282886)
Supplement: Attachment — Submitted filename: Response to Reviewers.docx [file pone.0282886.s002.docx]

**A point-by-point response to the Editor and Reviewers’ comments/suggestions**

Journal Requirements:

Please review your reference list to ensure that it is complete and correct. If you have cited papers that have been retracted, please include the rationale for doing so in the manuscript text, or remove these references and replace them with relevant current references. Any changes to the reference list should be mentioned in the rebuttal letter that accompanies your revised manuscript. If you need to cite a retracted article, indicate the article’s retracted status in the References list and also include a citation and full reference for the retraction notice.

Additional Editor Comments:

Dear Dr Puri,
It’s nice to read your paper and I found few issues to be addressed before the publication. Please also refers to review comments too.
The paper is well written however there is still rooms for improvement in each and every section. Few typos have been seen along with fluency of language in some parts of the paper.

Response: Thank you for the suggestions. We have reviewed the paper in its entirety, revised the language, and fixed typos. Two co-authors of this paper who are native English speakers have carefully checked fluency in language and grammar.

I have few specific comments as below:

Reorganize the abstract. I did not see how many participants were included in the study and findings in terms of statistical terms such as aOR (CI), x%(a/b) etc.. It is better to state clearly about your research design. What is the consistency for calculating wealth quantile with NDHS?

Response: Thank you for these suggestions. We have added detail on sample numbers and descriptive findings and reorganized the abstract. It is challenging to add aORs for all of the significant predictors mentioned in the abstract; there are 3 separate models (for gestational age at presentation, denial of care, and birth subsequent to denial) and for each of the models there are several significant coefficients for levels of wealth, education, age, and other variables. Given space limitations and for clarity, we have decided not to include all of these aORs in the abstract.

Methods:
You did not mention about the sample size and study design clearly. Please make it clear.
Not reaching till the result section, I found it is confusing about how any sampling sites. Please make it clear throughout the methodology section.

Response: We clarified in the second paragraph of the methods section that we recruited participants from a total of 22 sites, including 14 original sites and 8 additional sites.

Results
Line 219- 1,841 (95.3% of eligible women), who are these eligible women and how many?

Response: 8,856 women were screened. We have clarified that 1,925 of those (21.8%) were eligible. Of the 1,925 eligible, 1,835 (95.3%) consented to participate and completed a baseline interview. The final analytic sample included 1668 participants. This should help clarify why Table 1 is based on data from the 1,835 who completed a baseline interview.

Revise the presentation of table and make them as per standards. Please differentiate between use of N and n. Make consistent throughout the tables. I did not see any significance of each 100% and N data in tables separately

Response: We provide frequencies and percentages for the total (N) sample and percentages for the subgroups <10 weeks gestation and >=10 weeks/Don’t know. We removed unnecessary columns.

Table 2. what does that mean Col % Col % Col %?

Response: We have simplified the table heading to include simply “%” instead of “Col %”. We have removed this from all tables. Please note that Table 4 has missingness.

Describe about mixed effect in methodology section and why does it happen?

Response: In the methods section, we have clarified the following: “we used bivariable and multivariable mixed-effects logistic models accounting for clustering at the facility level, given that patient characteristics and service provision protocols including denial of care may be more similar within a given facility.

Line 256- Referencing 1- is it an appropriate referencing style?

Response: This was a foot note – now we have deleted this.

Line 268- Make standard referring to table. Please review other paper for writing styles.

Response: We have made this correction.

I found analytical write up in result section is compromised. Please write the result section in more analytical and interpretative way.

Response: Thank you for your suggestion. We are trying to both communicate the quantitative findings while emphasizing the policy importance.

Found about P>z, can you please have mentioned about the use of P>z and its significant in methodology section.

Response: We changed the description to “P value”

Discussion:
More analytical presentation of result is required along with comparing with other literature.

Response: Very few studies have been conducted in this area to compare our study findings. However, we have added a few more references and compared the results where available.

6. Review Comments to the Author

Please use the space provided to explain your answers to the questions above. You may also include additional comments for the author, including concerns about dual publication, research ethics, or publication ethics. (Please upload your review as an attachment if it exceeds 20,000 characters)

Reviewer #1: There is a need to get the paper rechecked by a native English speaker.

Response: The manuscript was carefully re-read by the English speaking co-authors and edits were made.

Avoid using "our" in the write-up for example, in line 43 replace "our" with "a".

Response: We have removed all the “our”s that we could. One remained, referencing our previous work.

In the background section, there is a need to add relevant literature from international studies. This shall be done with an intention to provide a conceptual framework for the regression model used in this study. In other words, the set of independent variables should be clearly mapped with relevant literature (for example, which studies back the inclusion of wealth quintile as an independent variable).

Response: We appreciate this comment. In our design of the study, we relied on our previous studies both in the United States and Nepal. This expertise did not emerge from a conceptual framework but from empirical investigations.

Please add some limitations, and avenues for future work. The paper ends abruptly; please add some policy recommendations that can be linked directly with the analysis conducted in the current study.

Response: Thank you for this suggestion. We have added some limitations and strengths of the study (see lines 436-444). Lines 451-460 are about policy/program recommendations that have emerged from our study findings.

We have added few sentences about future work (see lines 461-464).

Reviewer #2: The objective of the study is clearly stated by the authors. The data is organized in an effective manner and the statistical analysis is conducted with a high level of proficiency. As a result, the paper has a significant impact on the healthcare system of Nepal.

Response: Thank you for this comment.
